# Alcohol screening and brief interventions for adults and young people in health and community-based settings: a qualitative systematic literature review

**DOI:** 10.1186/s12889-017-4476-4

**Published:** 2017-06-09

**Authors:** Jane Derges, Judi Kidger, Fiona Fox, Rona Campbell, Eileen Kaner, Matthew Hickman

**Affiliations:** 1 University of Bristol School of Social and Community Medicine, Avon, Bristol, UK; 2 Institute of Health and Society, Newcastle University, Baddiley-Clark Building, Richardson Road, Newcastle upon Tyne, NE2 4AX UK

## Abstract

**Background:**

Systematic reviews of alcohol screening and brief interventions (ASBI) highlight the challenges of implementation in healthcare and community-based settings. Fewer reviews have explored this through examination of qualitative literature and fewer still focus on interventions with younger people.

**Methods:**

This review aims to examine qualitative literature on the facilitators and barriers to implementation of ASBI both for adults and young people in healthcare and community-based settings. Searches using electronic data bases (Medline on Ovid SP, PsychInfo, CINAHL, Web of Science, and EMBASE), Google Scholar and citation searching were conducted, before analysis.

**Results:**

From a total of 239 papers searched and screened, 15 were included in the final review; these were selected based on richness of content and relevance to the review question. Implementation of ASBI is facilitated by increasing knowledge and skills with ongoing follow-up support, and clarity of the intervention. Barriers to implementation include attitudes towards alcohol use, lack of structural and organisational support, unclear role definition as to responsibility in addressing alcohol use, fears of damaging professional/ patient relationships, and competition with other pressing healthcare needs.

**Conclusions:**

There remain significant barriers to implementation of ASBI among health and community-based professionals. Improving the way health service institutions respond to and co-ordinate alcohol services, including who is most appropriate to address alcohol use, would assist in better implementation of ASBI. Finally, a dearth of qualitative studies looking at alcohol intervention and implementation among young people was noted and suggests a need for further qualitative research.

## Background

Alcohol consumption is associated with numerous adverse health practices and outcomes [1, 2]. Efforts to mitigate alcohol use, especially amongst young people is of particular concern for Public Health world-wide and has led to the development of a plethora of alcohol screening and brief interventions (ASBI) aimed at addressing the rise in alcohol-related ill health [3, 4]. These have been designed both for ease of application across varied health and social care settings, and to be cost-effective [5–7]. Preventive approaches can be effective in reducing risky drinking, particularly when applied as part of routine screening procedures in primary healthcare settings [1, 8–15]. However, there is a lack of evidence from social care and non-health settings where implementation of appropriate interventions have had more mixed results. For example, lack of skills and knowledge in implementing interventions, attitudes to alcohol use by health professionals, and queries as to its appropriateness in community settings have all been cited as barriers [16–25].

Linked to this, most ASBI approaches have been developed for use with adults but there is increasing recognition that addressing young people’s use of alcohol requires a different approach [26, 27], especially as young people are more likely to access community-based services, such as local government, social services, or private agencies, than health services [28]. The development of more youth-oriented approaches such as web-based interventions and Motivational Interviewing (MI) have shown some modest effects in reducing alcohol consumption amongst adolescents, but requires further study [29–32]. Johnson and colleagues (2010) reviewed the qualitative literature on alcohol screening and brief interventions used with both adults and young people and identified lack of resources and training compounded by heavy staff workloads as the main barriers to effective implementation. However, despite describing itself as a qualitative paper, just over half these studies were quantitative (*n* = 28/47), comprising surveys, questionnaires and RCTs. The authors excluded educational and school-based interventions, stating that this was due to guidance having only been recently introduced (413), which meant interventions for young people were not represented. Our intention here, is to focus exclusively on the qualitative literature to generate an understanding of the contemporary facilitators and barriers influencing the implementation of ASBI in both healthcare and community-based settings, and to attempt to capture qualitative literature on youth service settings which quantitative evidence indicates are the places that young people are more likely to access [33].

### Aim and review question

Our aim is to explore the experiences of professionals implementing ASBI with adults and young people in healthcare and community-based settings. We have focused on qualitative studies using interviews, observations and/or focus groups as the basis for their analysis. The review question was: ‘What are the experiences of professionals in healthcare and community-based settings, in implementing alcohol screening and brief interventions with adults and young people?’

## Methods

### Search strategies and selection criteria

Searches were conducted between April and July, 2016. Electronic searches were made through Medline on Ovid SP, PsychInfo, CINAHL, Web of Science, and EMBASE. Google Scholar, citation searches, and ‘pearl-growing’ search techniques were used; the latter using a key citation to locate relevant index terms that help expand the scope of the search, rather than search by cited authors alone. This helped extend broaden the range of citations and further identify related subjects and themes [34, 35]. Search terms were applied in the following order: ‘alcohol’, ‘implementation’, ‘qualitative’. A further search was made adding the terms: ‘adolescents’ and ‘barriers’; of which only ‘barriers’ yielded further papers.

**Fig. 1:**
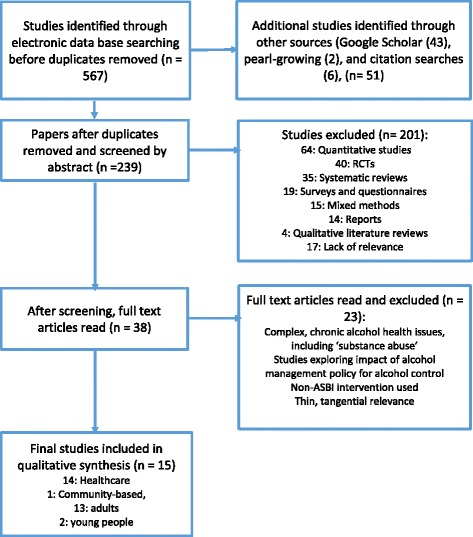
Inclusion & exclusion criteria

Articles were taken from international peer-reviewed journals, written or translated into English and published after 2000 to reflect contemporary findings. Qualitative studies were selected that addressed alcohol and brief screening interventions used with adults and young people, in both healthcare and community-based settings. Studies that were excluded, were: randomised trials, reports and surveys; alcohol with other forms of drug use; interventions that did not involve alcohol screening and brief intervention; interventions used with individuals who had complex long-term alcohol related disorders (see Fig. 1). There is a growing and important interest in digital interventions used with young people but so far, a lack of qualitative evaluation and therefore this is not included in the current review.

### Quality assurance

Selection criteria were based on clear evidence of methodological rigour, defined as: explanation of procedures used in the analysis, relevance of the sample group for our study, and a qualitative approach used in the analysis. Two researchers (JD and FF) cross-checked the final selected papers to ensure replicability and fit with our inclusion criteria.

### Analysis

Each of the included papers was read in full and a framework devised, which contained annotation of the content according to 1) strengths: richness and relevance, and weaknesses: thinness of data; and 2) tangential relevance to the review question. The following descriptors were used: study aims, methods and analysis used, strengths, weaknesses of the papers, and findings related to facilitators and barriers (see Table 1). Key concepts from the findings of each paper were extracted and examined closely for similarities and differences across all papers. From this, a set of themes were identified, which are reported on in the results section.

**Table 1:** A brief outline of selected papers

Authors & country	Study aims	Methods & analysis	Strengths	Weaknesses	Findings: facilitators	Findings: barriers
M Aalto, P Pekuri and K Seppa [47] Finland	Identification of obstacles for GPs^a^ and nurses in screening and brief intervention for heavy drinkers	*Method* Focus groups *Analysis* Content analysis	Innovative approach to looking at heavy drinking among patients	Lack of representativeness due to small numbers (18 GPs, 19 nurses) in one practice setting	Positive views about the need to address alcohol use with heavy drinking patients (and recognition that this extends to youth drinking)	Confusion over terms ‘early phase heavy drinking’, and ‘alcohol dependence’; pessimism about worth of addressing alcohol use; role responsibility ie addiction clinic better suited to address alcohol use; impact on doctor/ patient relationship; lack of guidelines
A Beich, D Gannik and K Malterud [49] Denmark	Qualitative study to explore GPs views of AUDIT in their daily practice	*Method* Interviews and focus groups *Theory/analysis* A ‘modified phenomenological’ approach	Included questions on use of alcohol screening with young people	Did not use transcriptions for coding, but direct from audio tapes, which might have lost some detail despite being used ‘to minimise loss of shades of meaning’	One doctor said he would try to incorporate the questionnaire into his practice	Doctors said they would not screen their patients for alcohol use, because: difficult to implement in normal flow of work; affected a ‘person-centred’ approach to patient interaction; additional workload
Broyles et al., (2012) [17] United States	Prospective study to identify the potential barriers and facilitators associated with nurse-delivered alcohol screening, BI^b^ and RT^c^ for hospital patients	*Method* Focus groups *Theory/ analysis*: from grounded theory	Early example of a study looking at professionals’ own alcohol consumption Discomfort identified in discussing alcohol in relation to age and sex of patients	Due to prospective nature of study, features were anticipated, rather than experienced in relation to barriers to implementation of SB and RT	Potential facilitators: development of knowledge, skills, communication and collaboration. Also expansion of roles in provision of care	Lack of alcohol-related knowledge and skills; poor communication across disciplines around alcohol-related care; poor alcohol assessment procedures and integration with e-records; concerns about negative reaction and limited motivation among patients; issues of compatibility in screening, BI and RT and healthcare philosophy and role; structural issues e.g. lack of time
N Fitzgerald, H Molloy, F MacDonald and J McCambridge [36] United Kingdom	To explore the impact of training for community-based staff in Scotland, in use of ABI^d^	*Method* Telephone interviews *Theory/ analysis* Thematic analysis	Wide variety of health and community-based staff were interviewed	Telephone interviews only Lack of clarity in defining the 3 themes related to barriers	Some increase in knowledge, or confidence in using the tool	Three themes identified: majority said they had not encountered appropriate clients with whom to use ABI; tool did not fit with practice or role; clients problems were too severe and therefore use of ABI was considered inappropriate
AJ Gordon, L Ettaro, KL Rodriguez, J Mocik and DB Clark [19] United States	Examines primary care providers, adolescents and parents attitudes to SBIRT in rural health setting	*Method:* Mixed methods study, including focus groups *Theory/ analysis:* Grounded theory, thematic analysis	Comprehensive exploration of professional, adolescent and parents attitudes to SBIRT	Limited to small rural area Limited focus on qualitative analysis of group interviews	All were enthusiastic about computer-based interventions Professionals and parents saw the benefits of SBIRT	Provider’s lacked training, tools and onward referral options; adolescents worried about confidentiality
Hutchings et al. (2006) [46] United Kingdom	To examine acceptability and feasibility of using SBI^e^ in primary care settings	*Method:* Focus groups *Theory/ analysis:* Framework analysis	Explored both patients’ and professionals’ diverse perspectives about who should implement ASBI in primary care setting	Small number of participants	Practice nurses seen as appropriate in addressing alcohol use, especially when ‘lifestyle’ issues needed to be raised	GPs and nurses: lack of awareness of importance of alcohol problems; ‘light’ drinkers considered more likely to benefit from SBI than ‘heavy’ drinkers; SBI should be addressed as ‘lifestyle’ intervention; worried about giving offence; work overload; young people’s alcohol use better addressed through educational institutions, not primary care
K Johansson, I Akerlind and P Bendtsen [41] Sweden	To identify to what extent nurses are willing to be involved in alcohol prevention	*Methods:* Focus group interviews *Theory/ analysis:* None mentioned	Addresses potential solutions from nurse perspective, in relation to screening strategies	Although a qualitative study, paper was written as a short communication and so lacked depth	Nurses felt they had learned new skills and had improved their skills in identifying risky drinking behaviour	Alcohol prevention seen as one among many interventions within role remit; preferred to screen only if a problem was identified first, and if onward referral services existed; worried about damaging relationship with patient; seen as ‘time consuming’; and lack of ‘self-efficacy’
M Keurhorst, M Heinen, J Colom, C Linderoth, U Mussener, K Okulicz-Kozaryn, J Palacio-Vieira, L Segura, F Silfversparre, L Slodownik, et al. [42] Catalonia, Netherlands, Poland, and Sweden	Looked at why screening not taking place with high risk patients ‘Why, how and for whom were interventions not given’ in 4 countries in Europe	*Method:* Semi-structured interviews *Analysis:* Thematic analysis	Example of newer method (Realist Evaluation) used to address the ‘how’ and ‘why’ questions regarding implementation process Unique exploration of use of financial re-imbursement of staff using ASBI	Professional backgrounds of participants were different within each country, making any generalisation to other settings difficult	Training and support improved knowledge, skills and prioritisation of alcohol as an issue Continuous provision, sufficient time to learn intervention techniques and tailoring to individual experience were helpful	Implementing electronic BI required more guidance than was available
CA Lock, E Kaner, S Lamont and S Bond [23] United Kingdom	Exploration nurses attitudes to brief screening and why it is underutilised in primary care	*Method:* semi-structured interviews *Analysis:* Grounded theory	Sets out a clear future agenda in terms of nurses involvement in alcohol-related interventions	Small sample size	Acknowledged importance of alcohol use as a health issue; could identify a need; perceived themselves as in best position to address alcohol use	Lack of training and preparation in alcohol intervention use; lack of confidence; lack of institutional support
C May, T Rapley and E Kaner [57] United Kingdom	To investigate how primary care practitioners were using aspects of brief interventions in their practice	*Method* Semi-structured interviews conducted in 2 phases *Analysis* Constant comparison	Useful exploration of context: contrast of experience-led vs protocol-led practices Discussed findings with participants which enhanced overall understanding	In exploring theoretically, the practice-research gap, there was less focus on recommendations for bringing the two components together less specific detail on how this might be promoted or supported for researchers	Practitioners own independent approaches to managing alcohol use amongst clients	Practice-research gap limited the acceptability of alcohol interventions
P Nygaard and OG Aasland [48] Norway	Qualitative study investigating barriers in implementing alcohol SBI amongst GPs	*Method*: Focus groups *Theory/ analysis:* Thematic analysis	Identified issue of prevention versus intervention	Focused on GPs exclusively Focused only on barriers, not facilitators Small numbers used in the study	If issue was about an intervention resulting from a recognized alcohol problem, GPs were more likely to use SBI Work-based health centres more likely to detect alcohol issues and intervene	Raising issue of alcohol due to ‘stigma’; integration into GPs daily practice; prevention vs. treatment conflict; organisational limitations; potentially negative impact on relationships with patients
AK Rahm, JM Boggs, C Martin, DW Price, A Beck, TE Backer and JW Dearing [2] United States	Evaluation of SAMHSA^f^ and SBIRT^g^ by mixed health-care practitioners	*Methods:* Focus groups and individual interviews *Analysis:* Content analysis	Early study eliciting patient views and perspectives of alcohol screening	Not generalisable to other studies where more limited resources might preclude use of clinical psychologists in implementing SBIRT	Psychologists effectively replaced nurses & doctors as screeners of alcohol use	Time limitations and prioritisation of other issues; organisational leadership was limited; training alone was not adequate – support of institution also recommended
CWM Tam, N Zwar and R Markham [44] Australia	To understand reasons for the low uptake of screening tools including AUDIT-C, among GPs	*Method*: Semi-structured group interviews *Analysis:* Grounded theory	Identifies the role of local context and socio-cultural perceptions of alcohol and its use	Small study and findings therefore limited Some barriers identified were specific to the Australian context ie cultural ideas around alcohol consumption, and therefore not generalisable	Detecting ‘at-risk’ drinking seen as important (but difficult)	Social and cultural barriers to asking about alcohol consumption; dynamics of patient-doctor interactions; alcohol screening questionnaires lack practical utility; community stigma and stereotypes of “problem drinking”; GP perceptions of unreliable patient alcohol use histories; and perceived threat to the patient-doctor relationship
AE Whittle, SM Buckelew, JM Satterfield, PJ Lum and P O’Sullivan [43] United States	To evaluate a curriculum, pre- and post-training, aimed at improving confidence of clinicians working with adolescents, using SBIRT & MI^h^	*Methods:* Mixed methods: questionnaire and observational study *Analysis:* Content analysis	Focus on evaluation of training, using information, workshop, observation of professionals using intervention with immediate feedback, and feedback from professionals after using intervention	Feedback given in writing, not verbally, which means some opportunities lost for further understanding and might have led to overvalued perspective	Improvement in skills; confidence in approaching alcohol use with young people; ability to self-reflect; opportunity to practice using interventions in training sessions	MI more time-consuming as an approach; knowing when to use MI or another approach, which might be more suitable
Williams, et al. (2016) [51] United States	To understand the process of implementation and ‘factors underlying quality problems’ in ASBI from the perspective of frontline staff in VA^i^ primary health care	*Method:* Semi-structured interviews *Analysis:* Template Analysis. Used to analyse qualitative data thematically by applying a coding ‘template’ [58]	Effective use of conceptual analytic framework - [59] and Mitchie (2005)	Site-specific limitations and therefore questionable generalizability to other settings	Staff considered alcohol use an important issue that required intervention within primary care settings	Implementation did not address training and infrastructure needs; lack of standardization; limited understanding of the goals of SBI; alcohol considered ‘specialists’ role; limited availability of treatment resources; negativity regarding patients’ interest in help-seeking

^a^GP General Practitioner

^b^BI Brief intervention

^c^RT Referral for Treatment

^d^ABI Alcohol Brief Intervention – term used in paper

^e^SBI is the term used in paper

^f^SAMHSA Substance Abuse & Mental Health Services Administration

^g^SBIRT Screening, Brief Intervention and Referral for Treatment

^h^MI Motivational Interviewing

^i^VA Veterans Health Administration

## Results

A preliminary examination of the literature found fewer studies focused on young people’s services and as a consequence, they are underrepresented in this synthesis. From 38 qualitative studies identified before final selection, only 6 were located in the community: one social care team [36], two from community pharmacy settings [37, 38], one school [39], and an indigenous community service in Australia [40]. A total of 567 articles were identified through the data base from their titles, and an additional 51 were found through Google Scholar, pearl and citation searches. After initial screening and removal of duplicates 239 remained. After all the abstracts had been read, 38 qualitative studies were selected for a fuller reading. Out of the 38 qualitative studies, 15 were selected for the final review based on an appraisal using CASP guidelines on study selection criteria (CASP, 2014), (see Fig. 1). From the 15 selected papers a number of intersecting themes emerged which highlighted some facilitators to implementation of ASBI, but a greater number of barriers. Implementation was facilitated by having adequate knowledge and expertise in screening and treating patients who present with alcohol issues. Generally, professionals acknowledged the importance of addressing alcohol as a public health concern but felt under-skilled and lacking in knowledge about alcohol and its impact on health. But even with training, there remained significant barriers to implementation and these fell into three key categories: 1) attitudes towards alcohol that affect how professionals address its use with patients; 2) organizational and structural barriers; and 3) training.

Two core themes emerged in relation to ASBI, namely; health and community-based professionals prospective views of the concepts, principles and processes of alcohol interventions; and secondly, professionals’ evaluation of the experience of implementation of ASBI. This helped capture any differences in hypothetical use of ASBI, and actual implementation with both adults and young people.

### Facilitators to implementation

#### Training

Of the 15 studies selected for review, there was a general appreciation amongst professionals that addressing alcohol use was an important aspect of healthcare delivery and that training was an important component of this. Training helped staff feel more confident and increased their knowledge and skills in relation to addressing alcohol use with both adults and young people [36, 41–43].

#### Screening measures

Certain aspects of specific tools were found helpful e.g. screening questions, AUDIT-C [44] and the ‘simplicity’ of an SBI tool was also valued, as was the opportunity to gain new knowledge and improve skills [42, 45]. Whilst this helped increase self-confidence, it did not necessarily translate into practice, rather most staff continued to find broaching the topic of alcohol with patients difficult, for reasons outlined below.

### Barriers to implementation

Overall, findings suggest that implementing ASBI in healthcare settings continues to be challenging. Barriers identified in this review, include: general attitudes towards alcohol and a lack of knowledge about its effects, and concerns about the effect on relationships with patients of addressing alcohol use. Professional roles and managing heavy workloads in the context of competing interests from other urgent health issues were also highlighted, and related to this, the lack of institutional support in implementing alcohol screening and brief interventions. One study noted that although nurses in primary care identified themselves as best suited to address alcohol use as part of a healthy lifestyle issue, this did not extend to young people whose alcohol use was considered more appropriately managed in an educational setting [46].

1. *Attitudes*
  A key concern amongst staff was the potential damage caused to relationships with patients by asking about alcohol use [41, 46–48]. Related to this, fears of stigmatizing or victimizing people unnecessarily was perceived to be detrimental to good practice [23, 44, 47, 48] and might offend or worse still, drive the patient away [41]. It was also described in 2 studies as interrupting the flow of interactions with patients both in terms of affecting doctor/ patient relationships, and getting in the way of completing busy ward schedules [17, 49]. Beich et al. (2002) found that doctors in primary care were generally against screening young people for hazardous drinking as their alcohol use was perceived as something that they would grow out of. They also suggested young people’s alcohol use should be addressed elsewhere, including in the family, and those doctors who recognized that addressing youth alcohol use was important, reported finding it difficult [49].
  Addressing alcohol issues was seen as hypocritical amongst some staff in relation to their own alcohol use, suggesting lack of awareness about safe drinking limits [17, 45]. In contrast, fears of becoming ‘moral guardians’ of their patients prevented some GPs discussing alcohol use [48], whilst other professionals felt that addressing alcohol use was unlikely to be beneficial due to a lack of motivation among patients [17, 47]. Patients interviewed in two studies did not however, report any concerns about alcohol being raised by their healthcare provider’s and in fact thought it was helpful [2, 17]. This shows important differences between patient and provider concerns and has implications for training.
  Professionals’ lack of confidence or ‘self-efficacy’ [41] was highlighted in several studies; for example, worries about a lack of experience and knowledge concerning the impact of alcohol on health, which then impaired the ability to address it with confidence. A number of studies found that raising alcohol use ‘cold’ i.e. without a clear reason or indicator, was a disincentive to talking about alcohol use with patients. Three studies suggested that a specialist or ‘lifestyle’ worker (as opposed to an ‘alcohol’ worker which was stigmatizing) would be more acceptable [2, 17, 46, 47]. Likewise, some felt that ‘specialist’ skills were required which may highlight a training need or workload issue. This is contradicted by nurses who identified their role as compatible with addressing alcohol as part of a lifestyle question, although not, as stated above, with young people. This indicates uncertainty about who is best placed to address alcohol use and how this should be done, specifically with young people. The difficulty in raising alcohol use was also linked to social and cultural attitudes; for example, in settings where alcohol is perceived as a social ‘norm’, talking about alcohol use was seen as hypocritical when it exists in a cultural setting where drinking alcohol is accepted as a pleasurable activity [44, 50]. Similarly, a study looking at implementation of SBIRT within rural primary care services in the US, found that parent’s attitudes were a barrier to addressing alcohol use with young people as parents were often providers of alcohol to their children, seeing it as part of a social ‘norm’. For community-based professionals in Scotland, the ASBI tool was not being used despite training, because the client group’s use of alcohol was considered either too severe; especially when complex mental health needs were also present, or not severe enough. Although many professionals did ask about alcohol use, they sometimes used their own strategies; not those of the ASBI tool. [36]. This was a similar finding from May et al. (2006) who found that GPs were already asking about alcohol use using approaches incorporated over long-standing practice.
2. *Institutional support*
  Most of the studies mentioned the lack of structural and organisational support acting as a barrier to implementing ASBI. This related to insufficient time allocated to conduct an intervention; especially lengthier approaches such as Motivational Interviewing commonly used with young people. Also highlighted was the lack of clarity in identifying the appropriate person to address alcohol use, prioritisation of other issues before alcohol use, and poor organisational leadership was mentioned. Addressing alcohol use with patients, was perceived as an additional burden on an already overloaded workforce and gave rise to the question of role responsibility as mentioned above, and whether a ‘specialist’ was required [46]. One study found that a brief intervention that worked by electronic prompts for staff to screen presenting patients, were not being implemented systematically because it was considered too impersonal and worked against person-centred care within the setting of a Veterans Administration (VA) hospital [51]. An aspect highlighted across several studies was the lack of anywhere to refer people on if alcohol was identified as a problem [19, 41, 51], which resulted in a reluctance to screen and intervene. Again, this raises an important issue in relation to ASBI used in healthcare settings where resources are stretched and in some cases, absent. Broyles noted that poor integration of services and the lack of proper assessment procedures negated the use of ASBI among nurses in the US and similarly, guidelines on how to implement ASBI were lacking for GPs working in Finland [47]. It was noted that while professionals understand and appreciate the need for ASBI, these kinds of institutional or organizational barriers can prevent its use. This becomes especially pertinent where staff report uncertainty about addressing young people’s alcohol use. Role responsibilities are also mentioned several times by nurses, who report a lack of interest among physicians in addressing alcohol use and, at the same time, suggest that allocation of resources should be directed towards providing specialist services [17].
3. *Training*
  Most studies found that professionals responded positively to the aims of training in ASBI and felt that it was an important area about which they needed to know more in order to gain confidence in using the tools. However, training was often lacking and even where it existed, there was evidence that training did little to change practice [2, 23, 42]. A number of factors were highlighted, as mentioned above, suggesting that training in ASBI per se, was not the problem but rather the context into which it was being applied. In Gordon et al.’s (2011) study concerning young people’s primary care services in the US, training in how to detect and intervene in alcohol use was lacking, especially in relation to parent’s attitudes towards drinking, which highlights important cultural and contextual differences [19]. The length of training also appeared to be a factor; ongoing post-training support, which was focused and relevant to the setting was perceived to be especially important [23]. The impact of training when provided only once was noted to be short-lived and insufficient in maintaining confidence [2, 25, 42]. The lack of training also links to both a lack of confidence in using ASBI and an absence of wider institutional support. So, whilst training itself had the potential to facilitate ASBI delivery, the lack of, or a failure to follow-up on training given did have a negative impact on implementation.

## Discussion

This is the first qualitative systematic literature review of the international literature examining the barriers and facilitators to healthcare and community-based professionals delivering ASBI to adults and young people. Findings indicate that most health professionals acknowledge the importance of addressing alcohol use among service users and value the increase in knowledge and expertise that training provides, but barriers remain to prevent the effective implementation of ASBI, including with younger age groups. These follow interconnected themes: attitudes towards alcohol use and how to address this in a way that is acceptable to both professionals and patients; and lack of organizational and structural support for implementation of ASBI for busy, overworked staff where other health problems compete for priority [2, 31, 41, 48, 52–55]. The review also found that training is generally perceived to be important and useful; the variety of methods used in training were well received and, importantly, improved confidence in addressing alcohol with young people [43]. However, one off training did not necessarily facilitate implementation, due to the need for ongoing follow-up and institutional support.

Attitudes to alcohol is a complex area in which disparate views influence whether or not staff are willing or feel able to raise the subject of alcohol use. The ‘moral’ dimension to asking about alcohol use points to alcohol’s social acceptability. In particular practitioners’ own alcohol use led to feelings of hypocrisy when raising it as a problem, which is in contrast to other behaviour such as illicit drug use. This concern over raising alcohol use with patients is exacerbated by work pressures which then feed into these attitudes and encourage the view that ‘someone else’ should be dealing with the problem which, feeds into the lack of training and institutional support. These inter-connecting barriers are especially the case in addressing alcohol use with young people; staff lack of confidence is manifested by their recommendation of ‘specialist’ services to deal with young people’s alcohol use. This suggests that barriers to implementing ASBI are not isolated issues, but are interlinked and need to be addressed as a whole: training, institutional support and more generally, attitudes to alcohol use among adults and young people. Interestingly, one study found that psychologists successfully replaced nurses in the implementation of ASBI, suggesting the potential for other professions to be involved in the implementation process [2]. But whether using psychologists is an effective use of resources, both professionally and economically, requires careful consideration.

Alcohol interventions used with young people do not feature extensively in the literature suggesting it is an area that requires further study. Of the two studies that were identified, one looked at the training clinicians received in AUDIT [49], and found that staff appreciated the skills and added confidence training provided but noted the lack of onward referral options, and the length of time involved in conducting Motivational Interviewing with young people [43]. The other study looked at training of GPs in SBIRT and Motivational Interviewing [43], but found that many worried that the computerised set-up of AUDIT was not compatible with the ‘person-centred’ approach they used with younger age groups. Young people were worried mostly about issues of confidentiality but were otherwise positive about the intervention [19]. This was a similar finding to adult populations in the studies reviewed, and is particularly noteworthy as it suggests a discrepancy between how ASBI is perceived by providers and recipients.

There was also a dearth of studies looking at community-based interventions (6/38 qualitative studies, and 1/15 of the final review papers) suggesting the urgent need for further investigation.

## Implications

New ASBI interventions are required that address institutional priorities, and workforce attitudes as part of the training, and that can demonstrate how to successfully integrate ASBI within health and community-based services with young people. Based on efficacy trials, there is some evidence that ASBI is an effective intervention in reducing risky alcohol consumption among adults, however evidence is lacking with regard to its implementation feasibility with young people who are less likely to be picked up through healthcare services [31, 56]. This review shows that ASBIs are perceived by healthcare workers to be more challenging when used with young people than adults, but this view is not shared among either young people or adults who have received ASBI. Central to addressing this disparity therefore, will be raising the confidence and awareness of healthcare workers in how to address alcohol use with younger age groups.

## Limitations

This study did not attempt to assess or evaluate the efficacy of brief screening and intervention tools or approaches for alcohol problems per se, as this question has already been covered extensively in the literature. Instead the review focused on the perceived facilitators and barriers to the implementation of alcohol screening and brief interventions, and therefore only included qualitative studies that had examined the *process* of ASBI implementation, rather than outcomes. The lack of qualitative studies examining ASBI delivery with younger age groups, and in community settings, and from lower income countries meant that findings were limited regarding this particular area of interest.

## Conclusions

Training and organizational support are interdependent in the successful implementation of ASBI in healthcare and community-based settings. Professionals not only need to develop new knowledge and skills in understanding alcohol use and how to address it with adults and young people, but also need environments that support this work. In resource-poor settings this is an ongoing but important challenge. Implementation of ASBI in the studies reviewed was also limited by attitudes; some nurses did not see it as their responsibility to address alcohol issues and ‘lifestyle workers’ were mentioned as being more appropriate. Increasing resources to overstretched healthcare providers, providing access to ongoing and regular training in both delivering ASBI and information about why it is important would therefore lead to more successful implementation of ASBI in healthcare settings. Further research is needed into the specific issue of ASBI in community-based settings, which has particular relevance for younger people who are less likely to access primary healthcare. Current public health policy promotes the increased use of ASBI in healthcare and other settings that come into contact with people who may drink hazardously. Using different approaches will be key to engaging younger people who have different drinking practices to adults; for example, drinking less frequently but at higher intensity [7]. Interventions using digital applications may also have greater relevance for young people, but studies are lacking in this area currently.

## Abbreviations

ABI: Alcohol Brief Intervention
ASBI: Alcohol Screening and Brief Intervention
AUDIT-C: The Alcohol Use Disorders Identification Test - Consumption
BI: Brief Intervention
CASP: Critical Appraisal Skills Programme
GP: General Practitioner
MI: Motivational Interview
RCT: Randomised Control Trial
RT: Referral for Treatment
SAMHSA: Substance Abuse and Mental Health Administration
SBI: Screening and Brief Intervention
SBIRT: Screening, Brief Intervention and Referral for Treatment
VA: Veterans Administration
